# Robustification of Naïve Bayes Classifier and Its Application for Microarray Gene Expression Data Analysis

**DOI:** 10.1155/2017/3020627

**Published:** 2017-08-07

**Authors:** Md. Shakil Ahmed, Md. Shahjaman, Md. Masud Rana, Md. Nurul Haque Mollah

**Affiliations:** ^1^Lab of Bioinformatics, Department of Statistics, University of Rajshahi, Rajshahi 6205, Bangladesh; ^2^Department of Statistics, Begum Rokeya University, Rangpur, Rangpur 5400, Bangladesh

## Abstract

The naïve Bayes classifier (NBC) is one of the most popular classifiers for class prediction or pattern recognition from microarray gene expression data (MGED). However, it is very much sensitive to outliers with the classical estimates of the location and scale parameters. It is one of the most important drawbacks for gene expression data analysis by the classical NBC. The gene expression dataset is often contaminated by outliers due to several steps involved in the data generating process from hybridization of DNA samples to image analysis. Therefore, in this paper, an attempt is made to robustify the Gaussian NBC by the minimum *β*-divergence method. The role of minimum *β*-divergence method in this article is to produce the robust estimators for the location and scale parameters based on the training dataset and outlier detection and modification in test dataset. The performance of the proposed method depends on the tuning parameter *β*. It reduces to the traditional naïve Bayes classifier when *β* → 0. We investigated the performance of the proposed beta naïve Bayes classifier (*β*-NBC) in a comparison with some popular existing classifiers (NBC, KNN, SVM, and AdaBoost) using both simulated and real gene expression datasets. We observed that the proposed method improved the performance over the others in presence of outliers. Otherwise, it keeps almost equal performance.

## 1. Introduction

Classification is a supervised learning approach for separation of multivariate data into various sources of populations. It has been playing significant roles in bioinformatics by class prediction or pattern recognition from molecular OMICS datasets. Microarray gene expression data analysis is one of the most important OMICS research wings for bioinformatics [1]. There are several classification and clustering approaches that have been addressed previously for analyzing MGED [2–11]. The Gaussian linear Bayes classifier (LBC) is one of the most popular classifiers for class prediction or pattern recognition. However, it is not so popular for microarray gene expression data analysis, since it suffers from the inverse problem of its covariance matrix in presence of large number of genes (*p*) with small number of patients/samples (*n*) in the training dataset. The Gaussian naïve Bayes classifier (NBC) overcomes this difficulty of Gaussian LBC by taking the normality and independence assumptions on the variables. If these two assumptions are violated, then the nonparametric version of NBC is suggested in [12]. In this case the nonparametric classification methods work well but they produce poor performance for small sample sizes or in presence of outliers. In MGED the small samples are conducted because of cost and limited specimen availability [13]. There are some other versions of NBC also [14, 15]. However, none of them are so robust against outliers. It is one of the most important drawbacks for gene expression data analysis by the existing NBC. The gene expression dataset is often contaminated by outliers due to several steps involved in the data generating process from hybridization of DNA samples to image analysis. Therefore, in this paper, an attempt is made to robustify the Gaussian NBC by the minimum *β*-divergence method within two steps. At step-1, the minimum *β*-divergence method [16–18] attempts to estimate the parameters for the Gaussian NBC based on the training dataset. At step-2, an attempt is made to detect the outlying data vector from the test dataset using the *β*-weight function. Then an attempt is made to propose criteria to detect the outlying components in the test data vector and the modification of outlying components by the reasonable values. It will be observed that the performance of the proposed method depends on the tuning parameter *β* and it reduces to the traditional Gaussian NBC when *β* → 0. Therefore, we call the proposed classifier as *β*-NBC.

An attempt is made to investigate the robustness performance of the proposed *β*-NBC in a comparison with several versions of robust linear classifiers based on M-estimator [19, 20], MCD (Minimum Covariance Determinant), and MVE (Minimum Volume Ellipsoid) estimators [21, 22], Orthogonalized Gnanadesikan-Kettenring (OGK) estimator including MCD-A, MCD-B, and MCD-C [23], and Feasible Solution Algorithm (FSA) classifiers [24–26]. We observed that the proposed *β*-NBC outperforms existing robust linear classifiers as mentioned earlier. Then we investigate the performance of the proposed method in a comparison with some popular classifiers including Support Vector Machine (SVM),* k*-nearest neighbors (*K*NN), and AdaBoost; those are widely used in gene expression data analysis [27–29]. We observed that the proposed method improves the performance over the others in presence of outliers. Otherwise, it keeps almost equal performance.

## 2. Methodology

### 2.1. Naïve Bayes Classifier

The naïve Bayes classifiers (NBCs) [30] are a family of probabilistic classifiers depending on the Bayes' theorem with independence and normality assumptions among the variables. The common rule of NBCs is to pick the hypothesis that is most probable; this is known as the maximum a posteriori (MAP) decision rule. Assume that we have a training sample of vectors {**x**_*jk*_ = (*x*_1*jk*_, *x*_2*jk*_,…,*x*_*pjk*_)^*T*^; *j* = 1, 2,…, *N*_*k*_} of size *N*_*k*_ for *k* = 1,2,…, *K*, where **x**_*ijk*_ denotes the* j*th observation of the* i*th variable in the* k*th population/class (*C*_*k*_). Then the NBCs assign a class label $\hat{y}=C_{k}$ for some* k* as follows:

(1) y^argmaxk∈1,…,K⁡ pCkfxjk ∣ θk,  Ck=argmaxk∈1,…,K⁡ pCk∏i=1pfxijk ∣ θk,Ck.

For the Gaussian NBC, the density function *f*_*k*_(**x**_*jk*_∣***θ***_*k*_, *C*_*k*_) of* k*th population/class (*C*_*k*_) can be written as

(2) fxjk ∣ θk,Ck=2π−p/2Λk−1/2·exp⁡−12xjk−μkTΛk−1xjk−μk,

where ***θ***_*k*_ = {***μ***_*k*_, Λ_*k*_}, and here ***μ***_*k*_ = (*μ*_1*k*_, *μ*_2*k*_,…,*μ*_*pk*_)^*T*^, is the mean vector and the diagonal covariance matrix is

(3) $$\begin{array}{c} \Lambda_{k}=\left[\begin{array}{ccc} {\hat{\sigma }}_{1k}^{2} & \cdots & 0\\ \vdots & \ddots & \vdots \\ 0 & \cdots & {\hat{\sigma }}_{pk}^{2} \end{array}\right]=\mathrm{diag}\left(\;{\hat{\sigma }}_{1k}^{2},{\hat{\sigma }}_{2k}^{2},\ldots ,{\hat{\sigma }}_{pk}^{2}\;\right) \end{array}$$

### 2.2. Maximum Likelihood Estimators (MLEs) for the Gaussian NBC

We assume that the prior probabilities *p*(*C*_*k*_) are known and the maximum likelihood estimators (MLEs) ${\hat{\mu }}_{k}$ and ${\hat{\Lambda }}_{k}$ of ***μ***_*k*_ and Λ_*k*_ are obtained based on the training dataset as follows:

(4) μ^k=1Nk∑j=1Nkxjk,

(5) Λ^=1N∑k=1KNkΛ^k,

(6) Λ^k=diagσ^1k2,σ^2k2,…,σ^pk2,

where ${\hat{\sigma }}_{ik}^{2}=\left(1/N_{k}\right)\sum_{j=1}^{N_{k}}{\left(x_{ijk}-{\hat{\mu }}_{ik}\right)}^{2}$,  ${\hat{\mu }}_{ik}=\left(1/N_{k}\right)\sum_{j=1}^{N_{k}}x_{ijk}$, and *N* = ∑_*k*=1_^*K*^*N*_*k*_;  *i* = 1,2,…, *p*.

It is obvious from (1)-(2) that the Gaussian NBC depends on the mean vectors (***μ***_*k*_) and diagonal covariance matrix (Λ_*k*_); those are estimated by the maximum likelihood estimators (MLEs) as given in (4)–(6) based on the training dataset. Therefore, MLE based Gaussian NBC produces misleading results in presence of outliers in the datasets. To get rid of this problem, an attempt is made to robustify the Gaussian NBC by minimum *β*-divergence method [16–18].

### 2.3. Robustification of Gaussian NBC by the Minimum *β*-Divergence Method (Proposed)

#### 2.3.1. Minimum *β*-Divergence Estimators for the Gaussian NBC

Let *g*(**x**_*k*_) be the true density and *f*(**x**_*k*_∣***θ***_*k*_) be the model density for* k*th populations; then the *β*-divergence of two p.d.f can be defined by

(7) Dβgxk,fxk ∣ θk·∫1βgβxk−fβxk ∣ θkgxk−1β+1gβ+1xk−fβ+1xk ∣ θkdxk

for *β* > 0 and *D*_*β*_(*g*(**x**_*k*_), *f*(**x**_*k*_∣***θ***_*k*_)) ≥ 0. Equality holds if and only if *g*(**x**_*k*_) = *f*(**x**_*k*_∣***θ***_*k*_) for all **x**_*k*_. When *β* tends to zero, *β*-divergence reduces to Kullback Leibler (K-L) divergence; that is,

(8) limβ↓0⁡ Dβgxk,fxk ∣ θk=∫gxklog⁡gxkfxk ∣ θkdxk=DKLgxk,fxk ∣ θk.

The minimum *β*-divergence estimator is defined by

(9) θ^kagrminθk′⁡ Dβgxk,fxk ∣ θk′=argmaxθk′⁡1Nβ∑k=1Kfβxk ∣ θk′−1β.

For the Gaussian density ***θ***_*k*_ = {***μ***_*k*_, Λ_*k*_} and the minimum *β*-divergence estimators ${\hat{\mu }}_{k,\beta }$ and ${\hat{\Lambda }}_{k,\beta }$ for the mean vector ***μ***_*k*_ and the diagonal covariance matrix Λ_*k*_, respectively, are obtained iteratively as follows:

(10) μ^k,βr+1=∑j=1NkWβxjk ∣ μ^kr,Λ^krxjk∑j=1NkWβxjk ∣ μ^kr,Λ^krΛ^k,βr+1=1N∑k=1KNkdiagσ^1k,β2,σ^2k,β2,…,σ^pk,β2,

where

(11) σ^ik,β2=β+1∑j=1NkWβxjk ∣ μ^kr,Λ^krxijk−μ^ik,βr2∑j=1NkWβxjk ∣ μ^kr,Λ^kr,

(12) Wβxjk ∣ μ^kr,Λ^kr=exp⁡−β2xjk−μ^krTΛ^kr−1xjk−μ^kr.

The formulation of (10)–(12) is straightforward as described in the previous works [17, 18]. The function in (12) is called the *β*-weight function, which plays the key role for robust estimation of the parameters. If *β* tends to 0, then (10) are reduced to the classical noniterative estimates of mean and diagonal covariance matrix as given in (4) and (6), respectively. The performance of the proposed method depends on the value of the tuning parameter *β* and initialization of the Gaussian parameters ***θ***_*k*_ = {***μ***_*k*_, Λ_*k*_}.

#### 2.3.2. Parameters Initialization and Breakdown Points of the Estimates

The mean vector ***μ***_*k*_ is initialized by the median vector, since mean and median are same for normal distribution and the median (Me) is highly robust against outliers with 50% breakdown points to estimate central value of the distribution. The median vector of* k*th class/population is defined as

(13) xk,md=Mej=1,2,…,Nkx1jk,Mej=1,2,…,Nkx2jk,…,,Mej=1,2,…,NkxpjkT.

The diagonal covariance matrix Λ_*k*_ is initialized by the identity matrix (**I**). The iterative procedure will converge to the optimal point of the parameters, since the initial mean vector would belong to the center of the dataset with 50% breakdown points. The proposed estimators can resist the effect of more than 50% breakdown points if we can initialize the mean vector ***μ***_*k*_ by a vector that belongs to the good part of the dataset and the variance-covariance Λ_*k*_ by the identity (**I**) matrix. More discussion about high breakdown points for the minimum *β*-divergence estimators can be found in [18].

#### 2.3.3. *β*-Selection Using *T*-Fold Cross Validation (CV) for Parameter Estimation

To select the appropriate *β* by CV, we fix the tuning parameter *β* to *β*_0_. The computation steps for selecting appropriate *β* by* T*-fold cross validation is given below.

**Step 1 .**

Dataset *D*_*k*_ = {**x**_*jk*_; *j* = 1,2,…, *N*_*k*_} is split into *T* subsets; *D*_*k*_(1), *D*_*k*_(2),…, *D*_*k*_(*T*) where *D*_*k*_(*t*) = {**x**_*tk*_; *t* = 1,2,…, *N*_*tk*_} and ∑_*t*=1_^*T*^*N*_*tk*_ = *N*_*k*_.

**Step 2 .**

Let *D*_*k*_^*c*^(*t*) = {**x**_*sk*_∣**x**_*sk*_ ∉ *D*_*k*_(*t*), *s* = 1,2,…, *N*_*tk*_^*c*^ = (*N*_*k*_ − *N*_*tk*_)} for *t* = 1,2,…, *T*.

**Step 3 .**

Estimate ${\hat{\mu }}_{k,\beta }$ and ${\hat{\Lambda }}_{k,\beta }$ iteratively by (10) based on dataset *D*_*k*_^*c*^(*t*).

**Step 4 .**

Compute CV(t) using dataset *D*_*k*_(*t*), for $t=1,2,\ldots ,T\;\;CV_{k}(t)=L_{\beta_{0}}({\hat{\mu }}_{k,\beta },{\hat{\Lambda }}_{k,\beta }|{\;D}_{k}(t))$, where $L_{\beta_{0}}({\hat{\mu }}_{k,\beta },{\hat{\Lambda }}_{k,\beta }|D_{kt})=\left(1/\beta_{0}\right)[1-\left(1/N_{kt}\right){\left|{\hat{\Lambda }}_{k,\beta }\right|}^{-\;\beta_{0}/2\left(1+\beta_{0}\right)}\sum_{x_{kj}\in D_{kt}}W_{\beta_{0}}\left(x_{kj}|{\hat{\mu }}_{k,\beta },{\hat{\Lambda }}_{k,\beta }\right)]$.

**Step 5 .**

End.

Computed suitable *β by*

(14) $$\begin{array}{c} \hat{\beta }=\underset{\beta }{\mathrm{argmin}}\;D_{k,\beta_{0}}\left(\;\beta \;\right),\;k=1,2,\ldots ,K, \end{array}$$

where *𝔇*_*k*,*β*_0__(*β*) =  (1/*N*_*k*_)∑_*t*=1_^*T*^CV_*k*_(*t*).

If the sample size (*N*_*k*_) is small such that *N*_*tk*_^*c*^ = (*N*_*k*_ − *N*_*tk*_) < *p*, then* T* = *N*_*k*_ (leave-one-out CV) can be used to select the appropriate *β*. More discussion about *β* selection also can be found in [16–18].

#### 2.3.4. Outlier Identification Using *β*-Weight Function

The performance of NBC for classification of an unlabeled data vector **x** using (1) not only depends on the robust estimation of the parameters but also depends on the values of **x** weather it is contaminated or not. The data vector **x** is said to be contaminated if at least one component of **x** = {*x*_1_, *x*_2_,…, *x*_*p*_} is contaminated by outlier. To derive a criterion of whether the unlabeled data vector **x** is contaminated or not, we consider *β*-weight function (12) and rewrite it as follows:

(15) Wk,βx ∣ μ^k,β,Λ^k,β=exp⁡−β2x−μ^k,βTΛk,β−1x−μ^k,β;β>0.

The values of this weight function lie between 0 and 1. This weight function produces larger weight (but less than 1) if **x** ∈ *C*_*k*_ and smaller weight (but greater than 0) if **x** ∉ *C*_*k*_ or contaminated by outlier. Therefore, the *β*-weight function (15) can be characterized as

(16) Wk,βx ∣ μ^k,β,Λ^k,β=>ψk,if  x∈Ck,≤ψk,if  x∉Ck  or  x  is  outlying.

The threshold value *ψ*_*k*_ can be determined based on the empirical distribution of *β*-weight function as discussed in [31] and by the quantile values of $W_{k,\beta }\left(x|{\hat{\mu }}_{k,\beta },{\hat{\Lambda }}_{k,\beta }\right)$ for *j* = 1,2,…, *N*_*k*_ with probability

(17) $$\begin{array}{c} \mathrm{Pr}\left\{\;W_{k,\beta }\left(\;x_{kj}=x\;|\;{\hat{\mu }}_{k,\beta },{\hat{\Lambda }}_{k,\beta }\;\right)\leq \psi_{k}\;\right\}\leq \vartheta , \end{array}$$

where *ϑ* is the probability for selecting the cut-off value *ψ*_*k*_ and the value of *ϑ* should lie between 0.00 and 0.05. In this paper, heuristically we choose *ϑ* = 0.03 to fix the cut-off value *ψ*_*k*_ for detection of outlying data vector using (18). This idea was first introduced in [31].

Then the criteria whether the unlabeled data vector **x** is contaminated or not can be defined as follows:

(18) wβx∑k=1KWk,βxjk ∣ μ^k,β,Λ^k,β=≥ψ,if  x  is  not  outlying,<ψ,if  x  is  outlying,

where *ψ* = ∑_*k*=1_^*K*^*ψ*_*k*_.

However, in this paper, we directly choose the threshold value of *ψ* as follows:

(19) $$\begin{array}{c} \psi =\left(\;1-\eta \;\right)\underset{y\in D}{\min}{\;W}_{\beta }\left(\;y\;\right)+\eta \;\underset{y\in D}{\max}\;W_{\beta }\left(\;y\;\right). \end{array}$$

With heuristically *η* = 0.10, where *𝔇* is the training dataset including the unclassified data vector **x**, (19) was also used in the previous works in [16, 18] to choose the threshold value for outlier detection.

#### 2.3.5. Classification by the Proposed *β*-NBC

When the unlabeled data vector **x** is usual, the appropriate label/class of **x** can be determined using the minimum *β*-divergence estimators ${\hat{\theta }}_{k,\beta }=\left\{{\hat{\mu }}_{k,\beta },{\hat{\Lambda }}_{k,\beta }\right\}$ of $\theta =\{{\hat{\mu }}_{k},{\hat{\Lambda }}_{k}\}$ in the predicting equation (1). If the unlabeled data vector **x**  is unusual/contaminated by outliers, then we propose a classification rule as follows. We compute the absolute difference between the outlying vector and each of mean vectors as

(20) dk=absx−μ^k,β=dk1,dk2,…,dkpT;k=1,2,…,K.

Compute sum of the smallest* r *components of **d**_*k*_ as *S*_*kr*_ = *d*_*k*(1)_ + *d*_*k*(2)_ + ⋯+*d*_*k*(*r*)_, where *r = *round (*p*/2). Then the unlabeled test data vector **x** can be classified as

(21) $$\begin{array}{c} \hat{y}=\arg\underset{k}{\min}\;S_{kr}. \end{array}$$

If the outlying test vector  **x**  is classified in to class *k*, then its* i*th component is said to be outlying if *d*_*ki*_ > *S*_*kr*_  (*i* = 1,2,…, *p*). Then we update **x**  by replacing its outlying components with the corresponding mean components from the mean vector ${\hat{\mu }}_{k,\beta }$ of* k*th population. Let **x**^*∗*^ be the updated vector of **x**. Then we use **x**^*∗*^ instead of **x** to confirm the label/class of **x**  using (1).

## 3. Simulation Study

### 3.1. Simulated Dataset 1

To investigate the performance of our proposed (*β*-NBC) classifier in a comparison with four popular classifiers (KNN, NBC, SVM, and AdaBoost), we generated both training and test datasets from *m* = 2 multivariate normal distributions with different mean vectors (***μ***_*k*_, *k* = 1,2) of length *p* = 10 but common covariance matrix (Λ_*k*_ = Λ; *k* = 1,2). In this simulation study, we generated **N**_1_ = 40 samples from the first population and **N**_2_ = 42 samples from the second population for both training and test datasets. We computed the training error and test error rate for all five classifiers using both original and contaminated datasets with different mean vectors {(***μ***_1_, ***μ***_2_ = ***μ***_1_ + *t*); *t* = 0,…, 9}, where the other parameters remain the same for each dataset. For convenience of the presentation, we distinguish the two mean vectors in such a way in which the second mean vector is generated by adding* t* with each of the components of the first mean vector.

### 3.2. Simulated Dataset 2

To investigate the performance of the proposed classifier (*β*-NBC) in a comparison of the classical NBC for the classification of object into two groups, let us consider a model for generating gene expression datasets as displayed in Table 1 which was also used in Nowak and Tibshirani [32]. In Table 1, the first column represents the gene expressions of normal individuals and the second column represents the gene expressions of patient individuals. First row represents the genes from group A and second row represents the genes from group B. To randomize the gene expression, Gaussian noise is added from *N*(0, *σ*^2^). First we generate a training gene-set using the data generating model (Table 1) with parameters *d* = 5 and *σ*^2^ = 1, where *p*_1_ = 30 genes denoted by {*A*_1_, *A*_2_,…, *A*_30_} are generated for group **A** and *p*_2_ = 30 genes denoted by {*B*_1_, *B*_2_,…, *B*_30_} are generated for group **B** with *n*_1_ = 30 normal individuals and *n*_2_ = 30 patients (e.g., cancer or any other disease). Then we generate a test gene-set using the same model with the same parameters *d* = 5 and *σ*^2^ = 1 as before, where *p*_11_ = 30 genes denoted by {*A*_31_, *A*_32_,…, *A*_60_} are generated for group  **A** and *p*_22_ = 30 genes denoted by {*B*_31_, *B*_32_,…, *B*_60_} are generated for group  **B** with *n*_11_ = 25 normal individuals and *n*_22_ = 25 patients (e.g., cancer or any other disease).

### 3.3. Simulated Dataset 3

To demonstrate the performance of the proposed classifier (*β*-NBC) in a comparison of some other robust linear classifiers based on the robust estimators (MCD, MVE, OGK, MCD-A, MCD-B, MCD-C, and FSA) as mentioned earlier for the classification of object into different groups, we have generated the training and test datasets from *m* = 2, 3 multivariate normal distributions with variables* p* = 10, 5, respectively. We consider *n*_1_ = 40 and *n*_2_ = 35 (*n* = *n*_1_ + *n*_2_) samples from *m* = 2 different multivariate normal populations *N*_*p*_(**µ**_1_, Λ_1_) and *N*_*p*_(**µ**_2_, Λ_2_). Here **µ**_2_ = **µ**_1_ + Ω with Ω = 0,1,…, 10 such that **µ**_1_ = **µ**_2_ for Ω = 0; otherwise **µ**_1_ ≠ **µ**_2_, where the scalar number Ω is the common difference between two corresponding mean components of ***μ***_1_ and ***μ***_2_, respectively. Similarly, for generating the training and test datasets, we consider the *n*_1_ = 30, *n*_2_ = 30, and *n*_3_ = 30 (*n* = *n*_1_ + *n*_2_ + *n*_3_) samples from *m* = 3. It is carried out with different means and common variance-covariance matrix of multivariate normal populations *N*_*p*_(***μ***_1_, Λ_1_), *N*_*p*_(***μ***_2_, Λ_2_), and *N*_*p*_(***μ***_3_, Λ_3_). In this case we consider ***μ***_*k*_ = ***μ***_*k*_ + Ω with Ω = 0,1,…, 10 and *k* = 1,2, 3 such that ***μ***_1_ = ***μ***_2_ = ***μ***_3_ for Ω = 0; otherwise ***μ***_1_ ≠ ***μ***_2_ ≠ ***μ***_3_, where the scalar number Ω is the common difference among the corresponding mean components of ***μ***_1_, ***μ***_2_, and ***μ***_3_, respectively.

### 3.4. Head and Neck Cancer Gene Expression Dataset

To demonstrate the performance of the proposed classifier (*β*-NBC) in a comparison with four popular classifiers (KNN, NBC, SVM, and AdaBoost) with the real gene expression dataset, we considered the head and neck cancer (HNC) gene expression dataset from the previous work [33]. The term head and neck cancer denotes a group of biologically comparable cancers originating from the upper aero digestive tract, including the following parts of human body: lip, oral cavity (mouth), nasal cavity, pharynx and larynx, and paranasal sinuses. This microarray gene expression dataset contains 12626 genes, where 594 genes are differentially expressed and the rest of the genes are equally expressed.

## 4. Simulation and Real Data Analysis Results

### 4.1. Simulation Results of Dataset 1

**Figure 1:**
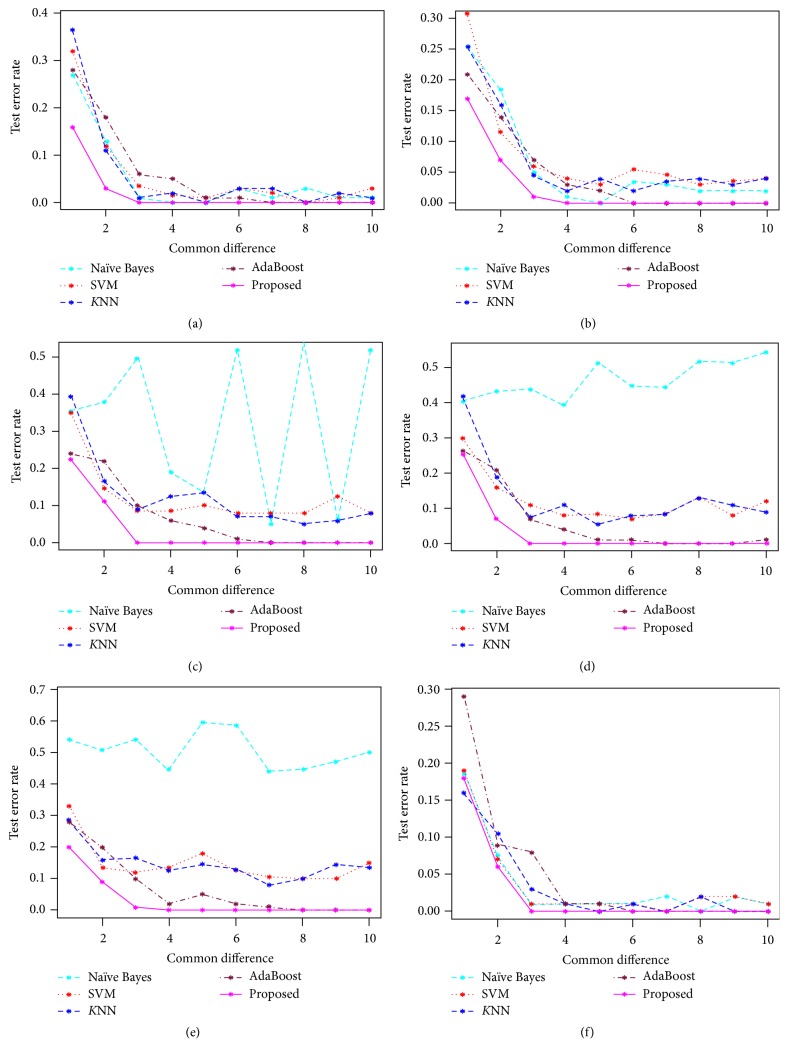
Misclassification error rate at different outlier levels: (a) 5% contamination rate, (b) 10% contamination rate, (c) 15% contamination rate, (d) 20% contamination rate, (e) 25% contamination rate, and (f) without contamination rate for the test dataset by the simulated dataset 1.

We have used the simulated dataset 1 to investigate the performance of the proposed method with the performance of the other popular classifiers such as classical NBC, SVM, KNN, and AdaBoost. Figures 1(a)–1(f) represent the test error rate estimated by these five classifiers against the common mean differences in absence of outliers (original dataset) and in presence of 5%, 10%, 15%, 20%, and 25% outliers in test dataset, respectively. From Figure 1(f) it is evident that in absence of outlier every method produces almost the same result, whereas, in presence of different levels of outliers (see Figures 1(a)–1(e)), the proposed method outperformed the other methods by producing low test error rate.  Table 2 is summarized with different performance measures (accuracy, sensitivity, specificity, positive predicted value (PPV), negative predicted value (NPV), prevalence, detection rate, detection prevalence, Matthews correlation coefficient (MCC), and misclassification error rate). All these performance measures are computed by the five methods (NBC, KNN, SVM, AdaBoost, and proposed).

From Table 2 we observed that the proposed method produces better results than the other classifiers (NBC, SVM, KNN, and AdaBoost), since it produces higher values of accuracy (>97%), sensitivity (>95%), specificity (>94%), PPV (>94%), NPV (>94%), and MCC (>94%) and lower values of prevalence and MER (<4%). The proportion test statistic [34] has been used to test the significance of several proportions produced by the five classifiers for each of the performance measures. The column 7 of Table 2 represents the *p* values of this test statistic. Since all the *p* values except MER are less than 0.01, so we can conclude that the performance results are highly statistically significant. The MER (*p* value < 0.05) is also statistically significant at 5% level of significance. So we may conclude from simulated dataset 1 that our proposed method performed better than the other classical methods for the contaminated dataset. It keeps equal performance in absence of outliers for the original dataset.

### 4.2. Simulation Results of Dataset 2

**Figure 2:**
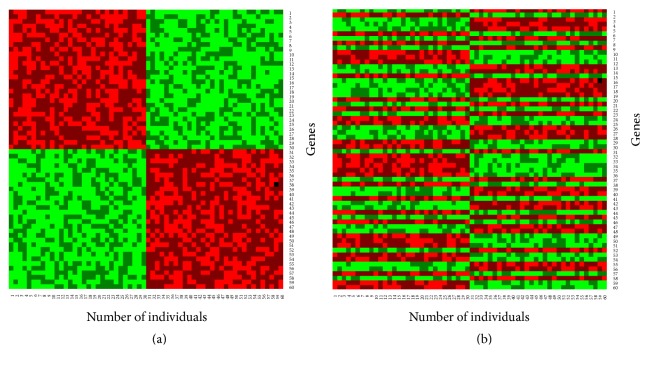
Simulation dataset 2 using data generating model (given in Table 1): (a) training gene-set and (b) test gene-set, without contamination.

**Figure 3:**
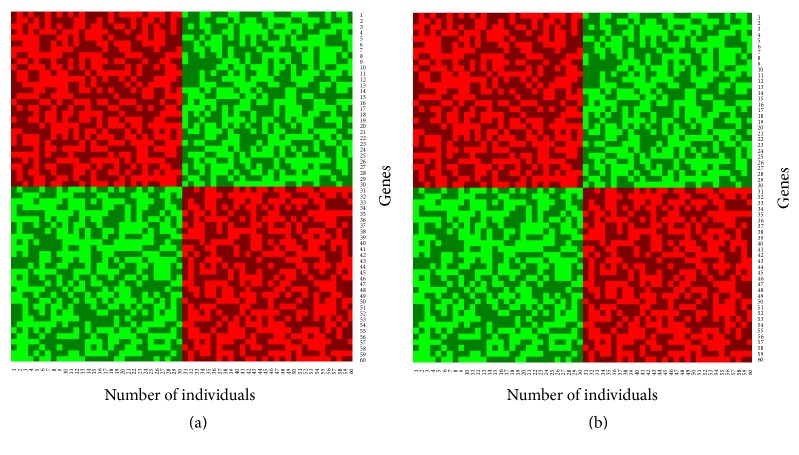
Classification results using (a) classical NBC and (b) proposed (*β*-NBC) method for the case without contamination based on the simulated dataset 2.

To investigate the performance of the proposed classifier (*β*-NBC) in a comparison of the classical NBC for the classification of objects into two groups, we considered the simulated dataset 2. Figures 2(a) and 2(b) show training and test datasets in absence of outliers, respectively. Here genes are randomly allocated in the test dataset. Figures 3(a) and 3(b) show the results of classified test dataset by classical and proposed NBC, respectively.

**Figure 4:**
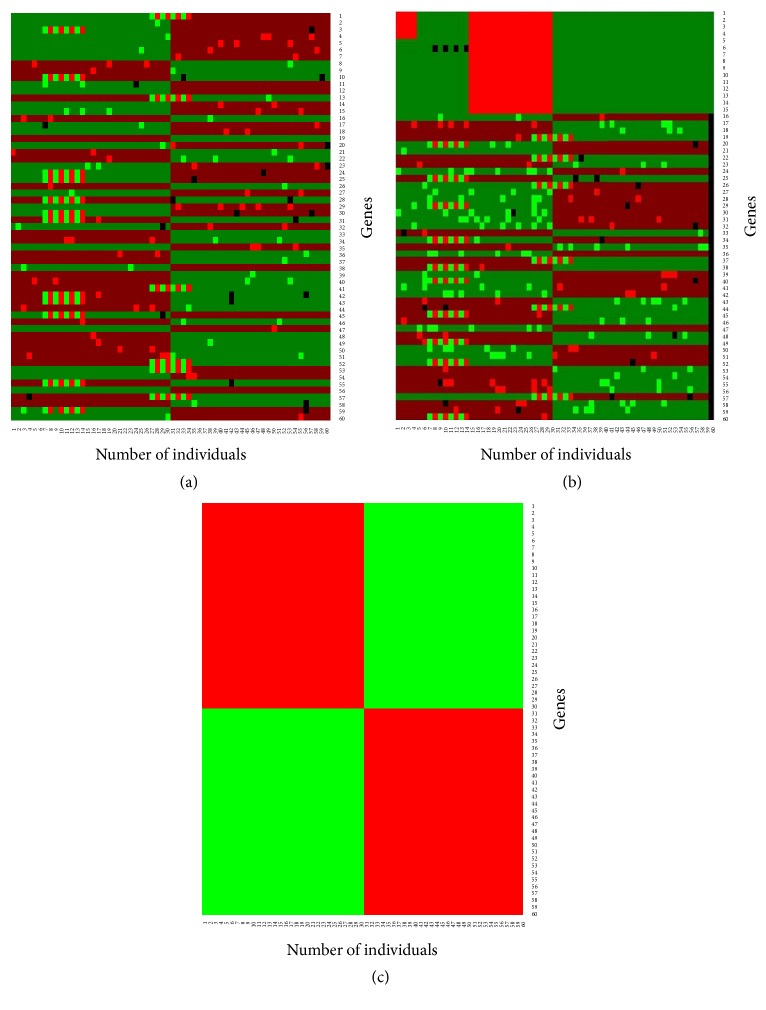
Classification results for the contaminated data: (a) contaminated test gene-set, (b) classified test gene-set by NBC, and (c) classified test gene-set by proposed method.

From classification results we observed that both the naïve Bayes procedures and proposed method produce almost the same results with low misclassification error rates in absence of outliers. To investigate the robustness performance of our proposed method in a comparison with the conventional naïve Bayes procedure for classification, we randomly contaminated 30% genes by outliers in the test gene-sets (Figures 4(a)–4(c)).

To classify sample into any one of the groups using the contaminated test gene-set (Figure 4(a)), we calculated the misclassification error rate by NBC and proposed method. From Figure 4 we see that the traditional naïve Bayes procedures fail to achieve correct classification (Figure 4(b)) and the misclassification error rate is 34%. Then we try to classify objects/patients using the proposed method which is shown in Figure 4(c). It is obvious from these figures that the classification performance of the proposed method is good and the misclassification error rate is approximately 5% for test gene datasets.

### 4.3. Simulation Results of Dataset 3

We also investigated the performance of the proposed robust naïve Bayes classifier in a comparison with classical naïve Bayes as well as robust linear classifier based on the MVE, FSA, MCD, MCD-A, MCD-B, MCD-C, and OGK estimators of the mean vectors and covariance matrices. We computed different performance measures such as average of true positive rate (TPR), false positive rate (FPR), area under the ROC curve (AUC), and partial AUC (pAUC) based on 50 replications of the dataset to measure the performance of all classifiers. A method is said to be better than others, if it produces larger values of TPR, AUC, and pAUC and smaller values of FPR and MER.

**Figure 5:**
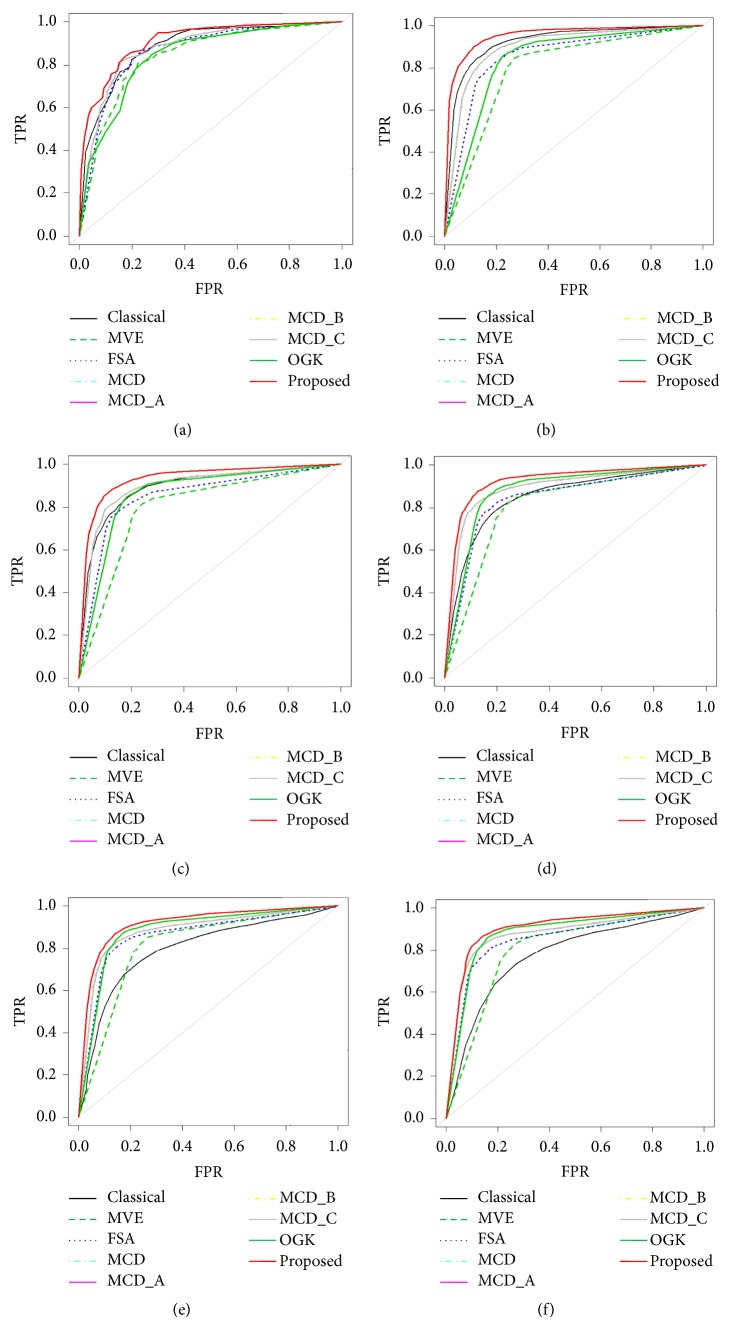
ROC curve for the 2- (two-) class classification of different estimators at different percentage of outliers: (a) absence of outliers, (b) 5% outliers, (c) 10% outliers, (d) 15% outliers, (e) 20% outliers, and (f) 25% outliers.

Table 3 shows the average values of AUC and pAUC at FPR = 0.2 based on the 50 replicated simulated datasets 3 with *p* = 15 for the two- (2-) class classification. The performance measures have been estimated by the classical, FSA, MCD, MVE, MCD-A, MCD-B, MCD-C, OGK, and proposed methods. They show the average estimates of AUC and pAUC for seven classifiers using simulated dataset 3 in absence and presence of outliers. We observed that in absence of outliers all the classifiers produce almost similar results. The proposed classifiers produced better result than the classical NBC and other robust estimators in presence of different levels (5%, 10%, 15%, 20%, and 25%) of outliers. Also MCD, MCD-A, MCD-B, and MCD-C show the constant performance result at the same level of outlier rate and varied for the different level of outlier rates. The ROC analysis also supported these results which are shown in Figures 5(a)–5(f), so we may conclude that the proposed method outperformed the others.

To investigate the performance of the proposed method in a comparison with other methods (classical, FSA, MCD, MVE, MCD-A, MCD-B, MCD-C, OGK, and proposed) for multiclass (3) classification problem. We generated simulated datasets 3 based on 50 replicated with *p* = 5 the number of variables. The performance measures were estimated for each of these methods.  Table 4 shows the average standard error of AUC and pAUC for multiclass classification. It is revealed that the proposed robust naïve Bayes classifier outperformed the classical and other robust linear classifiers in presence of outliers with false positive rate 0.2. The proposed method produces the larger values of AUC and pAUC and shows the lower values of MER and standard error of AUC and pAUC values. The performance measures using different types of MCD estimators were shown in the constant result at the same level of outlier rate. It was varied for the different levels of contamination rate.

### 4.4. Head and Neck Cancer Gene Expression Data Analysis

**Figure 6:**
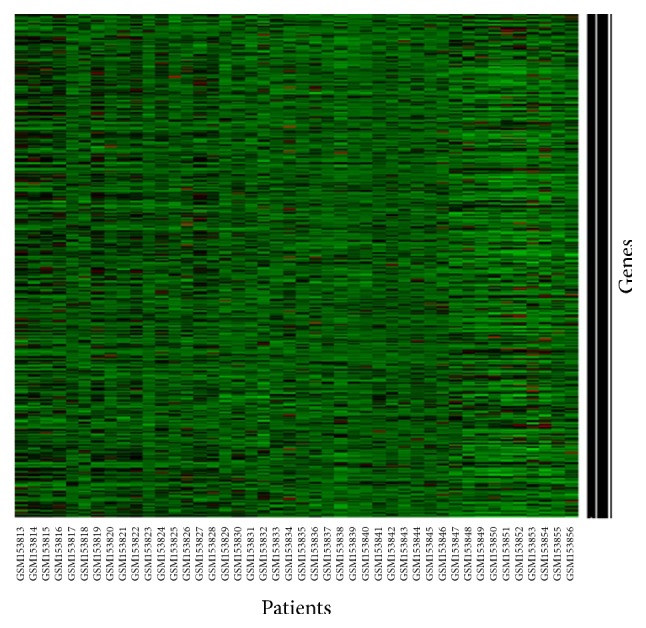
Differentially expressed genes of head and neck cancer dataset using bridge.

We also investigated the performance of the proposed method in real microarray gene expression dataset. The normalized Head and Neck cancer (HNC) dataset is considered here [33]. The RNA sample was extracted from the 22 normal and 22 cancer tissues for generating the HNC dataset. The Affymetrix GeneChip was used for processing RNA samples and finally got the quantified CELL file format. The Robust Multichip Analysis (RMA) and quantile normalization methods were used for processing the CELL files. The HNC dataset was 12,642 probe sets, 44 samples, and 42 significantly differentially expressed probe sets. The detailed discussion is shown in [33] for preprocessing of HNC dataset. We first select the differentially expressed (DE) genes whose posterior probability is more than 0.9; otherwise the genes are equally expressed (EE) using bridge R package [35] which is shown in Figure 6 that shows 594 differentially expressed genes from 12626 genes. We have performed the Anderson-Darling (A-D) normality test [36, 37] for the HNC dataset. The results show that a few numbers of DE genes (5%) for both normal and cancer groups break the normality assumption at 1% level of significance. Also we checked the independence assumption of DE genes using the mutual information [38]. We found that the mutual information for HNC dataset is 0.044 which is almost close to zero for both normal and cancer groups. So we may conclude that the DE genes almost satisfy the independence assumption. Therefore, we may assume that the HNC dataset almost satisfies the normality and independence assumption of NBC for a given class/groups.

**Figure 7:**
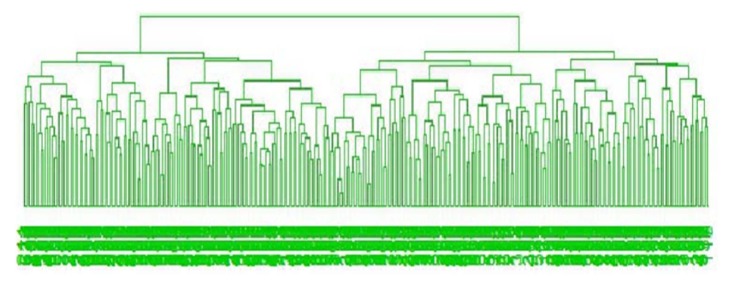
HC Dendrogram for calculated first half of DE genes.

**Figure 8:**
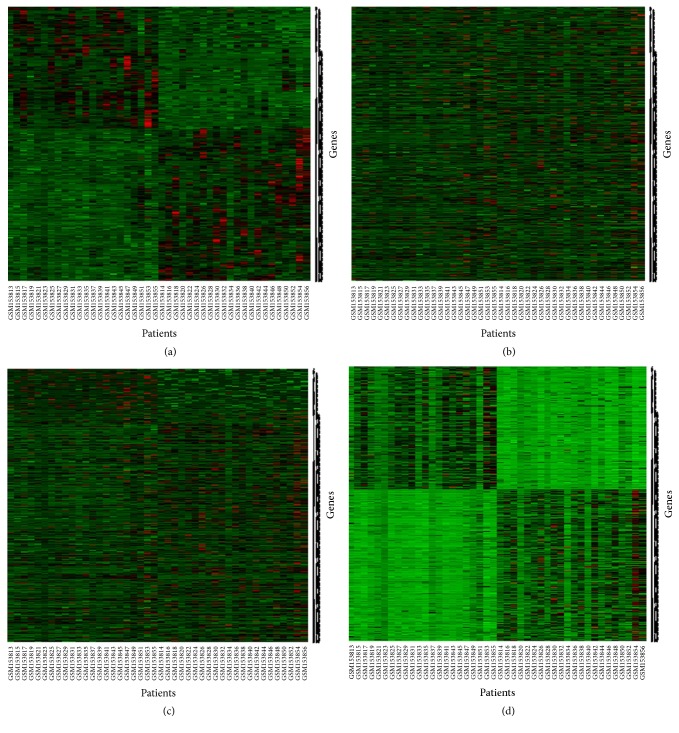
(a) Training gene data set; (b) test gene data set; (c) classification of gene data set by classical naïve Bayes procedure; (d) classification of gene data set by proposed (*β*-naïve Bayes) method.

For classification problem, we have considered half of the differentially expressed genes (594/2 = 297) as training gene-set and we identified their group using hierarchical clustering (HC).  Figure 7 represents the dendrogram of HC of half of the differentially expressed genes for training data. The rest of the 297 differentially expressed genes are considered as a test gene-set. Then we employed both classical NBC and robust NBC (*β*-NBC) in this dataset to classify cancer genes (see Figures 8(a)–8(d)). We observed that from Figure 8 the traditional naïve Bayes procedure can not find the group of gene properly whereas our proposed method (*β*-NBC) performs better for identifying the gene group in the HNC dataset.  Figure 8(d) shows that the proposed classifier shows better performance for classifying the samples than the classical method (Figure 8(c)).

We also computed different performance measures (accuracy, sensitivity, specificity, positive predicted value (PPV), negative predicted value (NPV), prevalence, detection rate, detection, prevalence, Matthews correlation coefficient (MCC), and misclassification error rate) by the five classification methods (NBC, KNN, SVM, AdaBoost, and proposed) using HNC dataset (Table 5). From Table 5 we have observed that the proposed classifier produces better results than the other classifiers (NBC, SVM, KNN, and AdaBoost). The proportion test [34] has shown that the *p* values <0.01 for the different performance results excluding MCC and MER. Then we may say that they are highly statistically significant. The MCC and MER are statistically significant at 5% level of significance because of the *p* values < 0.05. Hence, the performances of the proposed methods in real HNC data analysis are better than classical and other methods. Also this data set is contaminated by outliers reported in [31]. So we consider this dataset to investigate the performance of the proposed method in a comparison of some popular existing classifiers. We observed that the proposed method outperforms the others for this HNC dataset.

## 5. Discussion

In this paper, we discussed the robustification of Gaussian NBC using the minimum *β*-divergence method within two steps. For both simulated and real data analysis, at first, the mean vectors and the diagonal covariance matrices were computed by the minimum *β*-divergence estimators for the Gaussian NBC based on the training dataset. Then outlying test data vectors were detected from the test dataset using the *β*-weight function and outlying components in each test data vector were replaced by the corresponding values of their estimated mean vectors. Then the modified test data vectors were used as the input data vectors in the proposed *β*-NBC for their class prediction or pattern recognition. The rest of the data vectors from the test dataset were directly used as the input data vectors in the proposed *β*-NBC for their class prediction or pattern recognition. We observed that the performance of the proposed method depends on the tuning parameter *β* and the initialization of the Gaussian parameters. Therefore, in this paper, we also discussed the initialization procedure for the Gaussian parameters and the *β*-selection procedure using cross validation in Sections 2.3.2 and 2.3.3, respectively. The classifier reduces to the traditional Gaussian NBC when *β* → 0. Therefore, we call the proposed classifier *β*-NBC. We investigated the robustness performance of the proposed *β*-NBC in a comparison of several robust versions of linear classifiers based on MCD, MVE, and OGK estimators taking the smaller number of variables/genes (*p*) with larger number of patients/samples (*n*) in the training dataset, since these types of robust classifiers also suffer from the inverse problem of its covariance matrix in presence of large number of variables/genes (*p*) with small number of patients/samples (*n*) in the training dataset. We observed that the proposed *β*-NBC outperforms the existing robust linear classifiers as early mentioned in presence of outliers. Otherwise, it keeps almost equal performance. Then we investigated the performance of the proposed method in a comparison of some popular classifiers including Support Vector Machine (SVM),* K*-Nearest Neighbors (*K*NN), and AdaBoost which are widely used for gene expression data analysis [27–29]. In that comparison, we used both simulated and real gene expression datasets. We observed that the proposed method improves the performance over the others in presence of outliers. Otherwise, it keeps almost equal performance as before. The main advantage of the proposed classifier over the others is that it works well for both conditions of (i) *p* < *n* and (ii) *p* > *n*, and it can resist the effect of 50% breakdown points. If the dataset does not satisfy the normality assumptions, then the proposed method may show weaker performance than others in absence of outliers. However, the nonnormal dataset can transform to the normal dataset by some suitable transformation like Box-Cox transformation [39]. Then the proposed method would be useful to tackle the outlying problems. The proposed method may also suffer from the correlated observations. In that case, correlated observations can be transforming to the uncorrelated observations using standard principal component analysis (PCA) or singular value decomposition (SVD) based PCA. Then the proposed method would be more useful to tackle the outlying problems as before. However, in our current studied in this paper, we investigated the performance of the proposed classifier (*β*-NBC) in a comparison of some popular existing classifiers (NBC, KNN, SVM, and AdaBoost) including some robust linear classifiers (MCD, MVE, OGK, MCD-A, MCD-B, MCD-C, and FSA) using both simulated and real gene expression datasets, where simulated datasets satisfied the normality and independent assumptions. We observed that the proposed method improved the performance over the others in presence of outliers. Otherwise, it keeps almost equal performance. Usually gene expression datasets are often contaminated by outliers due to several steps involved in the data generating process from hybridization to image analysis. Therefore the proposed method would be more suitable for gene expression data analysis.

## 6. Conclusion

The accurate sample class prediction or pattern recognition is one of the most significant issues for MGED analysis. The naïve Bayes classifier is an important and widely used method for the class prediction in bioinformatics. However, this method suffers from outlying problems to estimate the location parameters in the MGED analysis. To overcome this we proposed *β*-NBC for estimating the robust location and scale parameters. In the simulation studies 1 and 2, we showed that, in presence of outliers, the proposed *β*-NBC outperforms other popular classifiers while datasets were generated from the multivariate and univariate normal distribution, respectively, and it keeps equal performance with the other classifiers, in absence of outliers. We also investigated the robustness performance of the proposed *β*-NBC in a comparison of linear classifier using some popular robust estimators in the simulation study 3. From this simulation study we observed that the proposed *β*-NBC outperforms existing robust linear classifiers. Finally we applied in the real HNC dataset; our proposed *β*-NBC showed better performance than the other traditional classifiers. Therefore, we may conclude that, in presence of outliers, our proposed *β*-NBC outperforms other methods using both simulated and real datasets.

## Supplementary Material

The R source code for the robust naive Bayes classifier (β-NBC). The robust classification of microarray gene expression data.

## Figures and Tables

**Table 1:** Gene expression data generating model.

Gene group	Individual	
	Normal	Patient
*A*	*d* + *N*(0, *σ*^2^)	−*d* + *N*(0, *σ*^2^)
*B*	−*d* + *N*(0, *σ*^2^)	*d* + *N*(0, *σ*^2^)

**Table 2:** Performance evaluation by different methods based on simulated dataset 1.

Prediction methods	NBC	SVM	*K*NN	AdaBoost	Proposed	*p* value
Accuracy	0.55	0.84	0.86	0.82	**0.97**	0.00
95% CI of accuracy	(0.45, 0.65)	(0.75, 0.90)	(0.77, 0.92)	(0.73, 0.89)	**(0.91**, **0.99)**	—
Sensitivity	0.54	0.78	0.79	0.90	**0.95**	0.00
Specificity	0.62	0.94	0.97	0.76	**0.94**	0.00
PPV	0.88	0.96	0.98	0.73	**0.94**	0.00
NPV	0.20	0.71	0.73	0.91	**0.94**	0.00
Prevalence	0.84	0.63	0.63	0.41	**0.40**	0.00
Detection rate	0.45	0.49	0.50	0.37	**0.48**	0.00
Detection prevalence	0.51	0.51	0.51	0.51	**0.51**	—
MCC	0.12	0.70	0.74	0.65	**0.94**	0.00
MER	0.49	0.18	0.17	0.08	**0.03**	0.03

**Table 3:** Performance evaluation of different methods using average values of AUC, pAUC, and standard error of pAUC for two-class classification based on simulated dataset 3.

Two- (2-) class classification				
Estimators	Average.AUCtest	SE.AUCtest	Average.pAUCtest	SE.pAUCtest
Without outliers				

Classical	0.88	0.01	0.11	0.01
MVE	0.84	0.04	0.10	0.03
FSA	0.86	0.04	0.11	0.03
MCD	0.88	0.04	0.12	0.02
MCD-A	0.88	0.04	0.12	0.02
MCD-B	0.88	0.04	0.12	0.02
MCD-C	0.88	0.04	0.12	0.02
OGK	0.85	0.05	0.10	0.02
Proposed	**0.91**	**0.01**	**0.13**	**0.02**

5% outliers				

Classical	0.92	0.03	0.14	0.01
MVE	0.79	0.07	0.04	0.04
FSA	0.85	0.06	0.10	0.05
MCD	0.90	0.06	0.13	0.04
MCD-A	0.90	0.06	0.13	0.04
MCD-B	0.90	0.06	0.13	0.04
MCD-C	0.90	0.06	0.13	0.04
OGK	0.84	0.04	0.07	0.04
Proposed	**0.95**	**0.02**	**0.16**	**0.01**

10% outliers				

Classical	0.89	0.03	0.13	0.02
MVE	0.80	0.05	0.05	0.05
FSA	0.85	0.06	0.11	0.04
MCD	0.90	0.04	0.13	0.02
MCD-A	0.90	0.04	0.13	0.02
MCD-B	0.90	0.04	0.13	0.02
MCD-C	0.90	0.04	0.13	0.02
OGK	0.86	0.05	0.10	0.05
Proposed	**0.93**	**0.02**	**0.15**	**0.01**

15% outliers				

Classical	0.85	0.05	0.11	0.03
MVE	0.81	0.05	0.06	0.05
FSA	0.84	0.06	0.10	0.04
MCD	0.89	0.05	0.13	0.03
MCD-A	0.89	0.05	0.13	0.03
MCD-B	0.89	0.05	0.13	0.03
MCD-C	0.89	0.05	0.13	0.03
OGK	0.88	0.05	0.10	0.05
Proposed	**0.92**	**0.03**	**0.14**	**0.02**

20% outliers				

Classical	0.92	0.02	0.14	0.01
MVE	0.75	0.05	0.016	0.03
FSA	0.81	0.06	0.06	0.06
MCD	0.87	0.04	0.12	0.02
MCD-A	0.87	0.04	0.12	0.02
MCD-B	0.87	0.04	0.12	0.02
MCD-C	0.87	0.04	0.12	0.02
OGK	0.78	0.05	0.02	0.05
Proposed	**0.95**	**0.01**	**0.17**	**0.00**

25% outliers				

Classical	0.81	0.07	0.09	0.03
MVE	0.72	0.08	0.04	0.04
FSA	0.83	0.07	0.08	0.05
MCD	0.86	0.07	0.11	0.05
MCD-A	0.86	0.07	0.11	0.05
MCD-B	0.86	0.07	0.11	0.05
MCD-C	0.86	0.07	0.11	0.05
OGK	0.87	0.04	0.11	0.043
Proposed	**0.92 **	**0.03**	**0.14**	**0.03**

**Table 4:** Performance evaluation of different methods using average values of AUC, pAUC, and standard error of pAUC using dataset 3 for multiclass (3) classification.

Multiclass (3) Class Classification				
Estimators	Average.AUCtest	SE.AUCtest	Average.pAUCtest	SE.pAUCtest
No outlier				

Classical	0.89	0.03	0.13	0.02
MVE	0.84	0.05	0.10	0.02
FSA	0.88	0.04	0.12	0.02
MCD	0.89	0.04	0.13	0.02
MCD-A	0.89	0.04	0.13	0.02
MCD-B	0.89	0.04	0.13	0.02
MCD-C	0.89	0.04	0.13	0.02
OGK	0.86	0.05	0.11	0.02
Proposed	**0.90**	**0.03**	**0.13**	**0.02**

5% outliers				

Classical	0.84	0.05	0.10	0.02
MVE	0.82	0.05	0.08	0.03
FSA	0.86	0.05	0.11	0.02
MCD	0.87	0.04	0.12	0.02
MCD-A	0.87	0.04	0.12	0.02
MCD-B	0.87	0.04	0.12	0.02
MCD-C	0.87	0.04	0.12	0.02
OGK	0.85	0.05	0.10	0.03
Proposed	**0.88**	**0.03**	**0.12**	**0.01**

10% outliers				

Classical	0.77	0.07	0.07	0.02
MVE	0.82	0.05	0.09	0.03
FSA	0.85	0.04	0.11	0.02
MCD	0.86	0.04	0.12	0.02
MCD-A	0.86	0.04	0.12	0.02
MCD-B	0.86	0.04	0.12	0.02
MCD-C	0.86	0.04	0.12	0.02
OGK	0.84	0.05	0.10	0.03
Proposed	**0.87**	**0.04**	**0.12**	**0.02**

15% outliers				

Classical	0.76	0.07	0.07	0.03
MVE	0.82	0.05	0.09	0.03
FSA	0.83	0.05	0.11	0.02
MCD	0.85	0.05	0.12	0.02
MCD-A	0.85	0.05	0.12	0.02
MCD-B	0.85	0.05	0.12	0.02
MCD-C	0.85	0.05	0.12	0.02
OGK	0.85	0.05	0.11	0.03
Proposed	**0.86**	**0.04**	**0.11**	**0.02**

20% outliers				

Classical	0.67	0.10	0.05	0.03
MVE	0.80	0.05	0.08	0.03
FSA	0.79	0.03	0.10	0.01
MCD	0.79	0.03	0.09	0.01
MCD-A	0.79	0.03	0.09	0.01
MCD-B	0.79	0.03	0.09	0.01
MCD-C	0.79	0.03	0.09	0.01
OGK	0.82	0.05	0.09	0.02
Proposed	**0.84**	**0.03**	**0.10**	**0.01**

25% outliers				

Classical	0.72	0.08	0.05	0.03
MVE	0.82	0.06	0.08	0.04
FSA	0.81	0.07	0.10	0.03
MCD	0.81	0.07	0.10	0.03
MCD-A	0.81	0.07	0.10	0.03
MCD-B	0.81	0.07	0.10	0.03
MCD-C	0.81	0.07	0.10	0.03
OGK	0.85	0.05	0.10	0.03
Proposed	**0.82 **	**0.05**	**0.10**	**0.02**

**Table 5:** Performance investigation using head and neck cancer data.

Prediction methods	NBC	SVM	*K*NN	AdaBoost	Proposed	*p* value
Accuracy	0.46	0.740	0.73	0.73	**0.76**	0.00
95% CI of accuracy	(0.35, 0.56)	(0.63, 0.81)	(0.63, 0.81)	(0.63, 0.81)	**(0.75**, **0.79)**	—
Sensitivity	0.46	0.79	0.84	0.79	**0.83**	0.00
Specificity	0.44	0.61	0.67	0.68	**0.77**	0.00
PPV	0.62	0.59	0.56	0.62	**0.86**	0.00
NPV	0.30	0.79	0.90	0.84	**0.87**	0.00
Prevalence	0.66	0.36	0.33	0.39	**0.43**	0.00
Detection rate	0.31	0.28	0.28	0.31	**0.43**	0.00
Detection prevalence	0.50	0.50	0.50	0.50	**0.50**	—
Balanced accuracy	0.45	0.71	0.76	0.74	**0.93**	0.00
MCC	−0.08	0.48	0.47	0.86	**0.90**	0.04
MER	0.50	0.27	0.25	0.08	**0.0**	0.05

## Additional Points

*Supplementary Materials.* The source code is written in R which is available in the Supplementary Material, available online at https://doi.org/10.1155/2017/3020627.

## References

[1] Veer V., Laura J. (2002). Gene expression profiling predicts clinical outcome of breast cancer. *Nature*.

[2] Dam S., Võsa U., van der Graaf A., Franke L., de Magalhães J. P. (2017). Gene co-expression analysis for functional classification and gene–disease predictions. *Briefings in Bioinformatics*.

[3] Yu X., Yu G., Wang J., Brock G. N. (2017). Clustering cancer gene expression data by projective clustering ensemble. *PLOS ONE*.

[4] Singh R. K., Sivabalakrishnan M. (2015). Feature Selection of Gene Expression Data for Cancer Classification: A Review. *Procedia Computer Science*.

[5] Novianti P. W., Jong V. L., Roes K. C., Eijkemans M. J. (2017). Meta-analysis approach as a gene selection method in class prediction: does it improve model performance? A case study in acute myeloid leukemia. *BMC Bioinformatics*.

[6] Chen M., Li K., Li H., Song C., Miao Y. (2017). The Glutathione Peroxidase Gene Family in Gossypium hirsutum: Genome-Wide Identification, Classification, Gene Expression and Functional Analysis. *Scientific Reports*.

[7] Jong V. L., Novianti P. W., Roes K. C. B., Eijkemans M. J. C. (2016). Selecting a classification function for class prediction with gene expression data. *Bioinformatics*.

[8] Buza K. (2016). Classification of gene expression data: a hubness-aware semi-supervised approach. *Computer Methods and Programs in Biomedicine*.

[9] Wang L., Oh W. K., Zhu J. (2016). Disease-specific classification using deconvoluted whole blood gene expression. *Scientific Reports*.

[10] Jiang L., Chen H., Pinello L., Yuan G.-C. (2016). GiniClust: Detecting rare cell types from single-cell gene expression data with Gini index. *Genome Biology*.

[11] Golub T. R., Slonim D. K., Tamayo P. (1999). Molecular classification of cancer: class discovery and class prediction by gene expression monitoring. *Science*.

[12] Soria D., Garibaldi J. M., Ambrogi F., Biganzoli E. M., Ellis I. O. (2011). A 'non-parametric' version of the naive Bayes classifier. *Knowledge-Based Systems*.

[13] Wright G. W., Simon R. M. (2003). A random variance model for detection of differential gene expression in small microarray experiments. *Bioinformatics*.

[14] Jiang L., Cai Z., Wang D., Zhang H. (2012). Improving Tree augmented Naive Bayes for class probability estimation. *Knowledge-Based Systems*.

[15] Balamurugan A. A., Rajaram R., Pramala S., Rajalakshmi S., Jeyendran C., Dinesh Surya Prakash J. (2011). NB+: An improved Naïve Bayesian algorithm. *Knowledge-Based Systems*.

[16] Mollah M. N. H., Minami M., Eguchi S. (2006). Exploring latent structure of mixture ICA models by the minimum *β*-divergence method. *Neural Computation*.

[17] Mollah M. N. H., Eguchi S., Minami M. (2007). Robust prewhitening for ICA by minimizing *β*-divergence and its application to FastICA. *Neural Processing Letters*.

[18] Nurul Haque Mollah M., Sultana N., Minami M., Eguchi S. (2010). Robust extraction of local structures by the minimum *β*-divergence method. *Neural Networks*.

[19] Randles R. H., Broffitt J. D., Ramberg J. S., Hogg R. V. (1978). Generalized linear and quadratic discriminant functions using robust estimates. *Journal of the American Statistical Association*.

[20] Maronna R. A. (1976). Robust *M*-estimators of multivariate location and scatter. *The Annals of Statistics*.

[21] Todorov V., Neykov P. Robust selection of variables in the discriminant analysis based on the mve and mcd estimators.

[22] Todorov V., Neykov N., Neytchev P. (1994). Robust two-group discrimination by bounded influence regression. A Monte Carlo simulation. *Computational Statistics and Data Analysis*.

[23] Todorov V., Pires A. M. (2007). Comparative performance of several robust linear discriminant analysis methods. *REVSTAT Statistical Journal*.

[24] He X., Fung W. K. (2000). High breakdown estimation for multiple populations with applications to discriminant analysis. *Journal of Multivariate Analysis*.

[25] Hubert M., Van Driessen K. (2004). Fast and robust discriminant analysis. *Computational Statistics & Data Analysis*.

[26] Hawkins D. M., McLachlan G. J. (1997). High-breakdown linear discriminant analysis. *Journal of the American Statistical Association*.

[27] Devi Arockia Vanitha C., Devaraj D., Venkatesulu M. (2015). Gene expression data classification using Support Vector Machine and mutual information-based gene selection. *Procedia Computer Science*.

[28] Parry R. M., Jones W., Stokes T. H. (2010). K-Nearest neighbor models for microarray gene expression analysis and clinical outcome prediction. *Pharmacogenomics Journal*.

[29] Long P. M., Vega V. B. (2003). Boosting and microarray data. *Machine Learning*.

[30] Friedman N., Geiger D., Goldszmidt M. (1997). Bayesian network classifiers. *Machine Learning*.

[31] Mollah M. M. H., Mollah N. H., Kishino H. (2012). *β*-empirical Bayes inference and model diagnosis of microarray data. *BMC Bioinformatics*.

[32] Nowak G., Tibshirani R. (2008). Complementary hierarchical clustering. *Biostatistics*.

[33] Kuriakose M. A., Chen W. T., He Z. M. (2004). Selection and validation of differentially expressed genes in head and neck cancer. *Cellular and Molecular Life Sciences*.

[34] Bergemann T. L., Wilson J. (2011). Proportion statistics to detect differentially expressed genes: A comparison with log-ratio statistics. *BMC Bioinformatics*.

[35] Gottardo R. (2017). *Bridge: Bayesian Robust Inference for Differential Gene Expression, R package version 1.40.0*.

[36] Anderson T. W., Darling D. A. (1952). Asymptotic theory of certain goodness of fit criteria based on stochastic processes. *Annals of Mathematical Statistics*.

[37] Thomas R., de la Torre L., Chang X., Mehrotra S. (2010). Validation and characterization of DNA microarray gene expression data distribution and associated moments. *BMC Bioinformatics*.

[38] Hausser J., Strimmer K. (2009). Entropy inference and the James-Stein estimator, with application to nonlinear gene association networks. *Journal of Machine Learning Research (JMLR)*.

[39] Box G. E. P., Cox D. R. (1964). An analysis of transformations. *Journal of the Royal Statistical Society. Series B*.

